# Anaerobic Spondylodiscitis caused by Parvimonas Micra in a Rheumatoid Arthritis Patient: Case Report and Review of the Literature

**DOI:** 10.31138/mjr.240823.asc

**Published:** 2023-08-24

**Authors:** Panagiotis Kalmoukos, Dimitrios Kouroupis, Georgios Sapouridis, Elisavet Simoulidou, Anna Varouktsi, Charalampos Zarras, Konstantinos Petidis, Athina Pyrpasopoulou

**Affiliations:** 1 2^nd^ Propaedeutic Department of Internal Medicine,; 2 Department of Radiology,; 3 Lab of Microbiology, Hippokration Hospital Thessaloniki, Thessaloniki, Greece

**Keywords:** anaerobic, spondylodiscitis, Parvimonas micra, rheumatoid arthritis, patients

## Abstract

We report the rare case of *Parvimonas micra* bacteraemia and secondary spondylodiscitis probably triggered by tooth injury in a rheumatoid arthritis patient. Anaerobic bacteria associated spondylodiscitis may evade diagnosis due to atypical clinical presentation usually lacking fever, and the difficulties related to microbiological characterisation of the pathogen. Even though anaerobic spinal infections may constitute <3% of the total, clinical suspicion should remain high, especially in the case of positive history for pre-existing oral cavity or gastrointestinal/gynaecological tract infections.

## INTRODUCTION

Pyogenic spondylodiscitis is a rare bacterial infection affecting more frequently the elderly.^1^ It’s pathophysiology is based on the inoculation of pathogenic bacteria either directly during spinal surgery or haematogenously, leading to the infectious degeneration of the vertebral body and intervertebral disc.^2^ Patients usually suffer from chronic comorbidities, such as diabetes, renal and hepatic failure, and immunosuppression, and are, more frequently, older men.^3^ The incidence of spondylodiscitis appears to have increased considerably in the recent years, peaking in people aged 75 years and older.^4^ Clinical presentation of pyogenic spondylodiscitis typically involves back pain or radiating pain, followed by fever, neurological deficits and systemic symptomatology (weight loss, night sweats, etc).^5^ Laboratory parameters, such as inflammatory markers (C-reactive protein) are elevated at presentation and their course tends to correlate with response to treatment.^6^ Microbiological diagnosis of the responsible pathogen is central to successful treatment and is associated with better clinical outcome.^7,8^ To enhance diagnostic yield, besides standard microbio-logical cultures, fine needle aspirates or core biopsies may be applied. Among common bacterial pathogens, *Staphylococcus aureu*s remains the most frequently identified microorganism; however, in elderly patients the incidence of gram negative bacteria – associated spondylodiscitis is increased compared to their younger counterparts.^9^ Anaerobic spondylodiscitis, despite rare, (accounting for less than 3% of pyogenic vertebral osteomyelitis cases) should always be taken into consideration and microbiologically pursued.^10^ We report here the rare case of *Parvimonas micra* induced pyogenic spondylodiscitis in a 68-year-old female rheumatoid arthritis patient after obtaining written informed consent.

## CASE REPORT

A 68-year-old female patient presented to the Emergency Department complaining of aggravating back pain after a fall occurring 20 days prior to admission, rendering her bed-bound. At the fall there was reportedly tooth fracture. She additionally reported gradual loss of appetite and weight loss.

The patient’s history was positive for rheumatoid arthritis under treatment with hydroxychloroquine and occasional use of injectable steroids, arterial hypertension, and a previous ischemic stroke without residual neurological deficits. One year previously the patient had suffered a similar fall which ended in spinal osteoporotic fractures in the lower thoracic spine. At the time the patient had received antiosteoporotic treatment because of severe vitamin D insufficiency.

**Figure 1.**
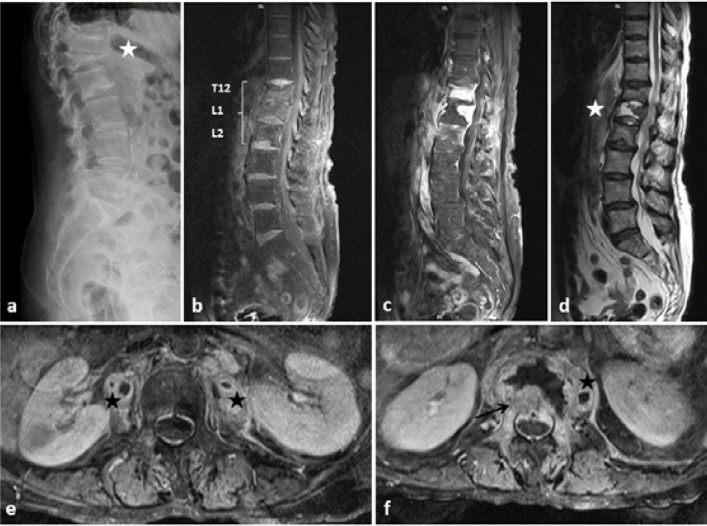
Imaging of the patient’s lumbar spine. **(A)** X-Ray of the lumbar spine showing compression of T12 and wedge-shaped deformity of L1 (white asterisk). **(B-D)** sagittal MRI lumbar spine. **(B)** T1-weighted MRI image of the lumbar spine and **(C)** fat-saturated T1-weighted MRI image (STIR) with post-contrast enhancement of the signal in the affected vertebrae and the surrounding tissues. **(D)** T2- weighted image (fluid signal - asterisk) differentiating the finding from metastatic lesions. **(E-F)** Axial MRI lumbar spine, black asterisks point to psoas abscesses. **(F)** Black arrow points to destruction of the vertebral body.

Upon admission the patient was normotensive, mildly tachycardic (90bpm), and afebrile. Physical examination was positive for severe pain, localized in the lower thoracic spine. The patient complained of mild radiation of the pain to the lower extremities and tenderness of the left knee, which pre-existed, and was attributed to the patient’s arthritis history. Laboratory tests upon admission revealed leucocytosis (White Blood Cells- WBC 16,500/ul – 86,6% Neutrophils-NE), and increased inflammatory markers (C-Reactive Protein [CRP] 149,8mg/L, normal values-nv <5). Chest-X-Ray and urinalysis were unremarkable. Urinary culture was negative. X-rays of the thoracic and lumbar spine showed old fractures of T12-L1. Two sets of blood cultures were taken upon admission and a third 48h later, when fever of 37.8°C was recorded. Two of the blood cultures grew *Parvimonas micra*. Initial antibiotic treatment (ciprofloxacin and teicoplanin) was modified to IV penicillin (16MU/d) and IV clindamycin (600mg qd) after completion of the microbiological analysis and sensitivity testing (bioMérieux Vitek 2 system according to the EUCAST sensitivity breakpoints). A magnetic resonance imaging of the lumbar spine was requested which revealed spondylodiscitis affecting T12-L2 vertebrae with abnormal enrichment in the surrounding paravertebral soft tissue and small abscess formation bilaterally within the psoas (**Figure 1**). A transthoracic cardiac ultrasound was performed without any evidence of endocarditis. Fine needle biopsy was not conducted due to the indicative clinical and radiological picture and the positive blood cultures in the absence of other primary foci. Subsequent blood cultures under treatment did not grow any pathogens. The patient responded rapidly clinically with concomitant normalisation of the laboratory tests and was released on oral clindamycin. Neurosurgeons consented for gradual mobilisation after release.

## DISCUSSION

Successful treatment of vertebral osteomyelitis depends on a variety of factors, most importantly prompt identification of the pathogen, early diagnosis, neurological deficits at diagnosis and underlying endocarditis.^11^ Anaerobic spondylodiscitis may indeed evade diagnosis, due to its atypical clinical presentation rarely accompanied by fever, the specific microbiological properties of the pathogens involved, and its radiological diversities.^12^ As in most musculoskeletal diseases, MRI remains the method of choice to distinguish between infectious degeneration of the vertebrae and intervertebral discs and other pathologies, eg, degenerative diseases, tumours, etc. Magnetic resonance imaging allows for the detection of bone oedema, an important early finding in vertebral osteomyelitis, while the administration of intravenous contrast agents allows for the detection of soft tissue infection and formed abscesses.^13^

*Parvimonas micra*, recently renamed from *Peptostreptococcus*, was first identified as the causative agent of spondylodiscitis in 1986.^14^ To identify cases of spondylodiscitis and/or arthritis reported in the literature, a literature search of PubMed and Scopus was performed using the keywords spondylitis, spondylodiscitis, arthritis, *Parvimonas* and *Peptostreptococcus*. A total of 31 cases were identified and summarised in **Table 1**.^14–33^ *Parvimonas micra* is a Gram positive, anaerobic coccus, which colonises the gastrointestinal tract and is frequently implicated in infectious complications of the oral cavity.^34^ The history of fall and tooth fracture may indeed have provided the source of bacteraemia in the case of our patient. Cases of *Parvimonas micra* bacteraemia and localised infections such as vertebral osteomyelitis are extremely limited; they mostly refer to elderly patients with comorbidities.^35^ The patient’s history of previous spinal trauma, rendering the vertebral column unstable and more susceptible to the inoculation of infectious agents mainly due to impaired local vascularity, and of rheumatoid arthritis/immunosuppression, conferred significant predisposition to the development of this condition.^36^ In patients with rheumatic conditions, especially in the absence of systemic symptomatology, such as fever, other inflammatory conditions of the spine have to be ruled out.^37^ The positive blood cultures and the detection of abscesses in the surrounding tissues clearly affirmed the infectious aetiology of the clinical condition. Outcome in the case of anaerobic joint and bone infections depends on the route of pathogen inoculation (postsurgical vs haematogenous spread), time to diagnosis, the presence of implants/osteosynthesis and the presence of neurological deficits at diagnosis. Besides targeted antibiotic treatment, IV for 2-4 weeks followed by oral treatment usually up to 3 months, a variety of supportive treatment strategies have been used, such as surgical debridement/decompression, and/or hyperbaric oxygen. Outcome is in the vast majority of cases favorable; relapses are associated with the site of infection and the presence of implanted foreign material.^38^

**Table 1.** Systematic review of reports of *Parvimonas micra* spondylodiscitis.

**Year of publication and author**	**# of cases**	**Gender, age (yrs)**	**Route of infection**	**Site of involvement**	**Co-morbidities**	**Treatment/duration**	**Outcome**
**Kalmoukos, current**	1	Female, 68	Haematogenous	T12-L2	Rheumatoid Arthritis Osteoporotic vertebral fractures T12-L1	Penicillin, clindamycin, 8wks	successful
**2020 Durovic^15^**	6	a.Male, 82 b.Male, 69 c.Male, 72 d.Female, 72 e.Male, 72 f.Female, 63	a.Haematogenous b.Unknown c Haematogenous d.Haematogenous e.Haematogenous f.Unknown	a.L1-L3 b.L2-L3 c.L1-L2 d.T12-L1 e.L4-L5 f. L2-L3	a.Renal failure Gout Previous spinal surgery: decompression L1–L5 b. Coronary heart disease Renal failure Diabetes mellitus II Previous spinal surgery: decompression and left discectomy L2-L3 c. Parkinson’s disease d. Metastatic breast cancer with diffuse vertebral metastases e. Diabetes mellitus II Previous spinal surgery: decompression L3-L4 f. None	a. Amoxicillin/clavulanic acid, meropenem, 5wks b. Amoxicillin/clavulanic acid, amoxicillin, 6wks c. Amoxicillin/clavulanic acid, penicillin, amoxicillin, 6wks d. Amoxicillin/clavulanic acid, moxifloxacin, 6wks e. Penicillin, ertapenem, clindamycin, 12wks f. Amoxicillin/clavulanic acid, amoxicillin, 6wks	a.death, unrelated b.partial improvement c.not reported d.Partial improvement e.successful f.partial improvement
**2019 Yoo^16^**	1	Female, 77	Haematogenous	L2-L3	Cerebrovascular accident, hypertension, hyperlipidaemia osteoporosis	Ceftriaxone, metronidazole 13wks	successful
**2018 van Duijvenbode^14^**	1	Male, 78	Unknown	L2-L3	Hypertension, ulcerative colitis, osteoarthritis	Penicillin, clindamycin, 6wks	successful
**2018 Mizuta^17^**	1	Female, 86	Unknown	L1-L2	Unknown	Metronidazole, 7wks	successful
**2017 Cleaver^18^**	1	Female, 45	Unknown	T12-L1	None, smoker	Imipenem/cilastatin, clindamycin, 6wks	successful
**2017 Higashi^19^**	1	Male, 67	Haematogenous	L4-L5	Diabetes mellitus 2	Ampicillin/sulbactam, ampicillin, 10wks	successful
**2015 Jones^20^**	2	a.Male, 72 b.Female, 72	a. Unknown b. Unknown	T12-L1 T5-T6	a. None b. Osteoarthritis	a. Piperacillin/tazobactam, amoxicillin/clavulanic acid, 8wks b. Piperacillin/tazoactam, 4wks	a.successful b.successful
**2015 George^21^**	1	Male, 49	Unknown	L3-L4	Spondylolisthesis and instrumented spinal fusion	Hardware explant + ceftriaxone-metronidazole, 6wks	successful
**2015 Gahier^22^**	3	a.Female, 59 b.Female, 82 c.Female, 60	a. Haematogenous b. Haematogenous c. Haematogenous	a.C4 b.T12-L1 c.L2-L3	a. None b. None c. None	a. Gentamicin/metronidazole/ amoxicillin, 14wks b. Ceftriaxone/gentamicin, amoxicillin 6wks c. Ceftriaxone/gentamicin, amoxicillin 12wk	a.successful b.successful c.successful
**2015 Endo^23^**	1	Female, 55	Unknown	L2-L3	None	Ampicillin-sulbactam, metronidazole, 10wks	not reported
**2015 Medina^24^**	1	Female, 23	Haematogenous	C6	None	Amoxicillin-clavulanic acid, rifampicin-clindamycin, 8wks	successful
**2015 Pilmis^25^**	1	Male, 83	Haematogenous	L4-L5	Previous hip/joint surgery, ischaemic heart disease	Amoxicillin-gentamicin/clindamycin rifampicin, 3,5mos	successful
**2015 Dahya^26^**	1	Male, 62	Endocarditis, haematogenous	L2-L3	Hepatitis C, liver transplantation, degenerative joint disease	Vancomycin, ceftriaxone	successful
**2014 Gonzalez^27^**	1	Male, 62	Unknown	T7-T8	Hypertension, Diabetes mellitus, cerebrovascular event	Clindamycin, 4mos	successful
**2014 Uemura^28^**	2	a.Male, 84 b.Female, 85	a. Unknown b. Haematogenous	c.L3-L4 d.T9-T10	a.Benign prostatic hypertrophy, periodontitis b. Hypertension, periodontitis	a. Ampicillin, sulbactam/amoxicillin, clavulanic acid 12wks b. ampicillin, amoxicillin, 12wk	a. successful b.successful
**2009 Fraisse^29^**	1	Male, 75	Haematogenous	L4-L5	Diabetes mellitus, hypercholesterolemia, tobacco and alcohol abuse	Amoxicillin+clavulanic acid+gentamicin, amoxicillin+clindamycin 12wks	successful
**2001 Brook^30^**	2	a. Male, 10 b. Male, 8	a. Unknown b. Unknown	a.T12-L1 b. L3-L4	a.None b. None	a. IV penicillin, oral amoxicillin b. Clindamycin	a. successful b. successful
**2000 Leder^31^**	1	Male, 70	Paraspinal abscess	L5-S1	Ulcerative colitis, osteoarthritis, benign prostatic hypertrophy	Penicillin, amoxicillin-metronidazole, 14wks	successful
**1998 Rousseau^32^**	1	Female, 82	Unknown	L3-L4	None	Amoxicillin	successful
**1986 Papasian^33^**	1	Male, 70	Unknown	L4-L5	Cataract operations, transurethral resection of the prostate, right inguinal herniorrhaphy.	Nafcillin-clindamycin, 6wks	successful

yrs; years, wks; weeks, mos; months, T: thoracic, L: lumbar, C: cervical.
